# 5-Fluorouracil-Related Pneumatosis Intestinalis: A Case Report and Review of the Literature

**DOI:** 10.7759/cureus.57576

**Published:** 2024-04-04

**Authors:** Muatassem A Alsadhan, Keely Kringlen, Meenu Singh

**Affiliations:** 1 Internal Medicine, University of Utah, Salt Lake City, USA; 2 Medicine, University of Utah, Salt Lake City, USA

**Keywords:** tongue scc, cytotoxic drugs, chemotherapy, pneumatosis intestinalis, 5-fluorouracil

## Abstract

Pneumatosis intestinalis (PI) refers to the presence of air within the bowel wall. It can be associated with many causes including chemotherapy. We report a case of a 70-year-old male with metastatic tongue squamous cell carcinoma (SCC), whose hospital course was complicated by diarrhea and the development of PI, which was attributed to 5-fluorouracil (5-FU) chemotherapy after a comprehensive diagnostic workup and reassuring physical examination. The patient was treated conservatively with antibiotics and a bowel rest. A repeat imaging done before discharge showed stable findings. The patient was discharged afterward without complications. We highlight the importance of recognizing 5-FU as a cause for PI among patients with reassuring physical examination and diagnostic workup. Furthermore, we highlight that it may still be successfully managed with conservative measures.

## Introduction

Cancer is a leading cause of mortality worldwide. With the increased use of chemotherapeutic agents and targeted therapies (TT), in addition to the overall complexity of cancer patients, many uncommon treatment-related side effects are being recognized. 5-Fluorouracil (5-FU) is an antimetabolite drug commonly used in gastrointestinal and head and neck cancers [1]. Its main side effects include nausea, vomiting, immunosuppression, and, uncommonly, pneumatosis intestinalis (PI). In this review, we present a case of an elderly patient whose hospital course was notable for 5-FU-related PI. Moreover, we discuss medication-related PI and its potential outcomes with a comprehensive presentation.

## Case presentation

A 70-year-old male with metastatic tongue squamous cell carcinoma (SCC), recurrent aspirations, and on gastrostomy tube feeding presented initially with encephalopathy, fever, and non-bloody diarrhea eight days following the first cycle of docetaxel (75 mg/m^2^), carboplatin (466 mg), and 5-FU (750 mg/m^2^ for four doses). His physical exam was notable for tachycardia (121 beats per minute), tachypnea (27 breaths per minute), fever (38.5 degrees Celsius), and altered mental status. His labs were notable for severe neutropenia with an absolute neutrophil count (ANC) of 400/uL. He was treated with cefepime (2 g every 12 hours), with an impression of febrile neutropenia secondary to gastrointestinal source or possible pneumonia with chest X-ray findings of left perihilar and retrocardiac opacities, although the results of the diagnostic tests were negative (blood cultures, urine cultures, stool culture, and Clostridium difficile testing). Abdominal imaging was not done at admission.

**Figure 1:**
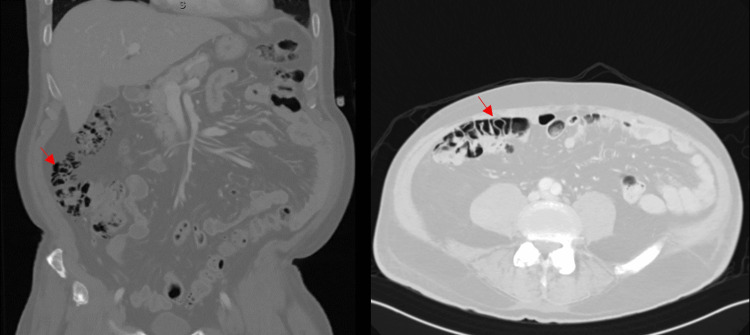
Left: coronal section with notable PI in the ascending colon (arrow). Right: cross-sectional abdominal CT with PI in the ascending colon (arrow).

By day five, the fever had resolved, and the patient’s mental and respiratory status improved; however, he had worsening diarrhea with the development of hematochezia, although he remained hemodynamically stable with an unremarkable abdominal examination, stable hemoglobin, liver and kidney function, and repeat negative infectious workup. A CT of the abdomen obtained was notable for the development of ascending and transverse colonic pneumatosis without bowel wall thickening, perforation, free fluid, abnormal distention, or signs of bowel ischemia (Figure 1).

Surgical consultation was obtained, which the patient and his family refused secondary to high-risk surgery with recent chemotherapy, and the patient overall felt well. The patient was managed conservatively with IV piperacillin-tazobactam (3.375 g every six hours), IV hydration, withholding enteral feedings, and serial abdominal exams that were unremarkable. His serial lactic acid normalized following a single elevated reading of 2.7 mmol/L (normal: 0.5-2.2 mmol/L). By day eight, the patient showed clinical improvement and was tolerating tube feeds without any issues. A repeat CT showed persistent PI in the ascending and transverse colon. The patient was resumed on tube feedings without complications. Colonoscopy was not pursued secondary to the risk of barotrauma.

The patient was discharged on day 13 on oral metronidazole (500 mg three times a day) and cefixime (400 mg daily). He was seen in the oncology clinic three days after discharge, was tolerating tube feeding, and was stable. 5-FU was not resumed on the next chemotherapy cycle. The patient was readmitted 14 days later with respiratory distress from enlarging necrotic lymph nodes in the neck and was found to have methicillin-sensitive staphylococcus aureus (MSSA) bacteremia. Despite aggressive ICU care, he passed away on the second day of hospitalization from respiratory failure and cardiac arrest.

## Discussion

PI is a radiological finding and not a diagnosis by itself. It refers to the abnormal collection of air within bowel walls. The overall incidence of PI in the general population has been reported to be 0.03% based on an autopsy series. It is typically seen with CT, which is the imaging study of choice [2]. PI could be primary or secondary. While primary PI refers to idiopathic PI, secondary PI can originate from many causes such as intestinal ischemia, gastrointestinal infections, endoscopic procedures including jejunostomy tubes and double contrast barium enema, collagen vascular diseases, inflammatory bowel disease, pulmonary diseases, or medications (e.g., steroids, immunosuppressants, alpha-glucosidase inhibitors, sorbitol, or lactulose) [2].

Depending on the cause, PI ranges in clinical presentation from a benign asymptomatic finding to a life-threatening condition. Factors including fever, sepsis, signs and symptoms of peritoneal irritation, lactic acidosis, bowel wall thickening, and pneumoperitoneum help in differentiating the benign PI from concerning cases. While the pathophysiology underlying PI is not completely understood, few theories exist in the literature. For instance, mechanical theory attributes the transmural translocation of air to be related to increased luminal pressure as in cases of obstruction for instance. Another theory is the bacterial theory, wherein the presence of air is because of gas production by intramural bacteria [2]. Several anticancer medications have been linked to PI including cytotoxic drugs such as cyclophosphamide, daunorubicin, cytarabine, 5-FU, and TT such as sunitinib, cetuximab, or bevacizumab [3]. Medication-related PI is speculated to be related to the loss of mucosal barrier integrity allowing gas-producing bacteria to enter the bowel wall, which can be exacerbated by the presence of neutropenia [4]. This also can be exacerbated by the cytotoxic effects of chemotherapy on the intestinal mucosa by reduction in proliferation and increase in apoptosis [1]. It is also thought that mesenteric microvascular disruption such as vasospasm, thrombosis, or shunting might be a contributing factor [5]. 

A recent systematic review published in 2022 reported approximately nine cases of 5-FU-associated PI and 88 cases associated with cancer treatment overall [3]. TT with monoclonal antibodies and kinase inhibitors accounted for 47 cases, while cytotoxic agents accounted for 37 of the cases. The remaining cases were related to immunotherapy. The median time to onset was six weeks of anticancer treatment [3]. PI was found to mostly localize to the colon, followed by the small intestine, followed by the entire intestinal tract in the least number of cases. PI was found to be fatal in 13% of cases (11/88), with others experiencing asymptomatic to mild courses [3]. The therapeutic approach to medication-related PI varied from a conservative approach to surgical emergency depending on clinical presentation and CT findings, with the hepatic, portal, and portomesenteric gas being the most concerning [2]. Gazzaniga et al. reported that the reintroduction of offending medication to the treatment regimen was not common (18/88) and was associated with a 33% chance of PI recurrence (6/18), with no fatal outcomes [3].

A major limitation presented in our case is the lack of initial abdominal imaging on admission and the lack of prompt initiation of antibiotics with anaerobic coverage such as metronidazole or piperacillin-tazobactam despite having non-bloody diarrhea upon presentation. It thus remains unclear if earlier imaging or anaerobic coverage would have affected patient outcomes.

To our knowledge, there are five case reports on 5-FU-related PI without concomitant TT use (Table 1) [1,6-9]. All patients were older than 60, and all but one were male. The main presenting symptoms were abdominal pain and diarrhea. Two out of five patients underwent surgical exploration, and the rest were treated conservatively, which usually referred to IV fluids, oxygen, bowel rest, and possibly antibiotics. There was no reported inpatient mortality, and all patients had improvement in PI before discharge. It remains unclear if 5-FU was later re-challenged in four out of these five cases.

**Table 1:** Reported cases of 5-FU-related PI without TT agents. Abd= Abdominal, NR= Not reported, SCC= Squamous cell carcinoma. *5-FU was used with leucovorin and no other chemotherapies. **The exact cycle length was not reported; however, each cycle of folinic acid, fluorouracil, and irinotecan (FOLFIRI) is typically two weeks [10].

Authors	Age and sex	Diagnosis	Chemotherapy agents besides 5-FU	Period after chemotherapy	Presenting symptoms	Main treatment	Rechallenged with 5-FU?
Vargas et al., 2016 [1]	65, M	Gastroesophageal cancer, stage IV	Cisplatin	After the fourth cycle. Each cycle is 21 days	Abd. pain, diarrhea, distension	Conservative	NR
Mimatsu et al., 2008 [6]	76, M	Rectal cancer, stage III	Leucovorin*	After the first cycle. Each cycle is one week	Abd. pain, diarrhea	Surgery	No
Vanderschueren et al., 2020 [7]	78, F	Gastric adenocarcinoma	Irinotecan	Six days after the second cycle of FOLFIRI**	Abd. pain, diarrhea	Conservative	NR
Kouzo et al., 2017 [8]	70, M	Esophageal cancer	Cisplatin	14 days	Abd. pain, distension	Conservative	NR
Ozturk et al., 2017 [9]	61, M	Nasopharyngeal cancer	Docetaxel	Seven days	Abd. pain, vomiting	Surgery	NR
Our case	70, M	Tongue SCC	Docetaxel, carboplatin	16 days	Diarrhea	Conservative	No

## Conclusions

All in all, 5-FU-related PI remains a rare diagnosis that carries a good in-hospital prognosis, and it may still be treated conservatively. Most providers are not aware of the association of medications with PI, including in tertiary cancer care cancers. In our case, we incidentally found literature supporting the role of 5-FU as a culprit agent, as our comprehensive workup did not support other etiologies. The lack of initial abdominal imaging and earlier initiation of an antibiotic with anaerobic coverage upon presentation was a major limitation in our case.

Because of a lack of literature, the optimal approach to medication-related PI remains unclear, particularly as it relates to the role of surgery, antibiotics, imaging surveillance, and the reintroduction of chemotherapy agents. In cases of benign PI, surgery may be harmful, as the disease usually resolves with conservative treatment.

## References

[1] Vargas A, Pagés M, Buxó E (2016). [Pneumatosis intestinalis due to 5-fluorouracil chemotherapy]. Gastroenterología y Hepatología (English Edition).

[2] Ho LM, Paulson EK, Thompson WM (2007). Pneumatosis intestinalis in the adult: benign to life-threatening causes. AJR Am J Roentgenol.

[3] Gazzaniga G, Villa F, Tosi F (2022). Pneumatosis intestinalis induced by anticancer treatment: a systematic review. Cancers (Basel).

[4] Pengermä P, Katunin J, Turunen A, Rouvelas I, Palomäki A, Kechagias A (2022). Is surgical exploration mandatory in pneumatosis intestinalis with portomesenteric gas? Lesson learned in a neutropenic patient under chemotherapy. ANZ J Surg.

[5] McGettigan MJ, Menias CO, Gao ZJ, Mellnick VM, Hara AK (2016). Imaging of drug-induced complications in the gastrointestinal system. Radiographics.

[6] Mimatsu K, Oida T, Kawasaki A, Kano H, Kuboi Y, Aramaki O, Amano S (2008). Pneumatosis cystoides intestinalis after fluorouracil chemotherapy for rectal cancer. World J Gastroenterol.

[7] Vanderschueren L, Coulier B (2020). Massive benign pneumatosis intestinalis. J Belg Soc Radiol.

[8] Kouzu K, Tsujimoto H, Hiraki S (2017). A case of pneumatosis intestinalis during neoadjuvant chemotherapy with cisplatin and 5-fluorouracil for esophageal cancer(†). J Surg Case Rep.

[9] Ozturk M, Camlidag I, Nural MS, Ozbalci GS, Bekci T (2017). A rare cause of acute abdomen in the ED: chemotherapy-induced pneumatosis intestinalis. Turk J Emerg Med.

[10] (2024). FOLFIRI for colon and colorectal cancer. https://www.chemoexperts.com/folfiri-folinic-acid-fluorouracil-irinotecan-colon-cancer.html.

